# Supplementary material for: Analysis of temporal trends in potential COVID-19 cases reported through NHS Pathways England
Source: Sci Rep. 2021 Mar 29;11:7106. doi: 10.1038/s41598-021-86266-3 (PMC8007605; doi:10.1038/s41598-021-86266-3)
Supplement: Supplementary file 1 — Supplementary Information [file 41598_2021_86266_MOESM1_ESM.pdf]

Analysis of temporal trends in potential COVID-19 cases reported through NHS Pathways England: Supplementary material

Quentin J. Leclerc, Emily S. Nightingale2, Sam Abbott, CMMID COVID-19 Working Group, Thibaut Jombart

Sensitivity analysis for rolling window model

A rolling window analysis allows us to better capture trends in the data, by reducing the impact of noise between different days on our estimates. However, the choice of the time window can affect our estimates. Using a 7 days window in our model leads to high variation between estimates (Figure S1A), while a 21 days window prevents us from capturing finer trends in the data (Figure S1B). We therefore chose to report results for a 14 days window as a compromise between these two sizes.

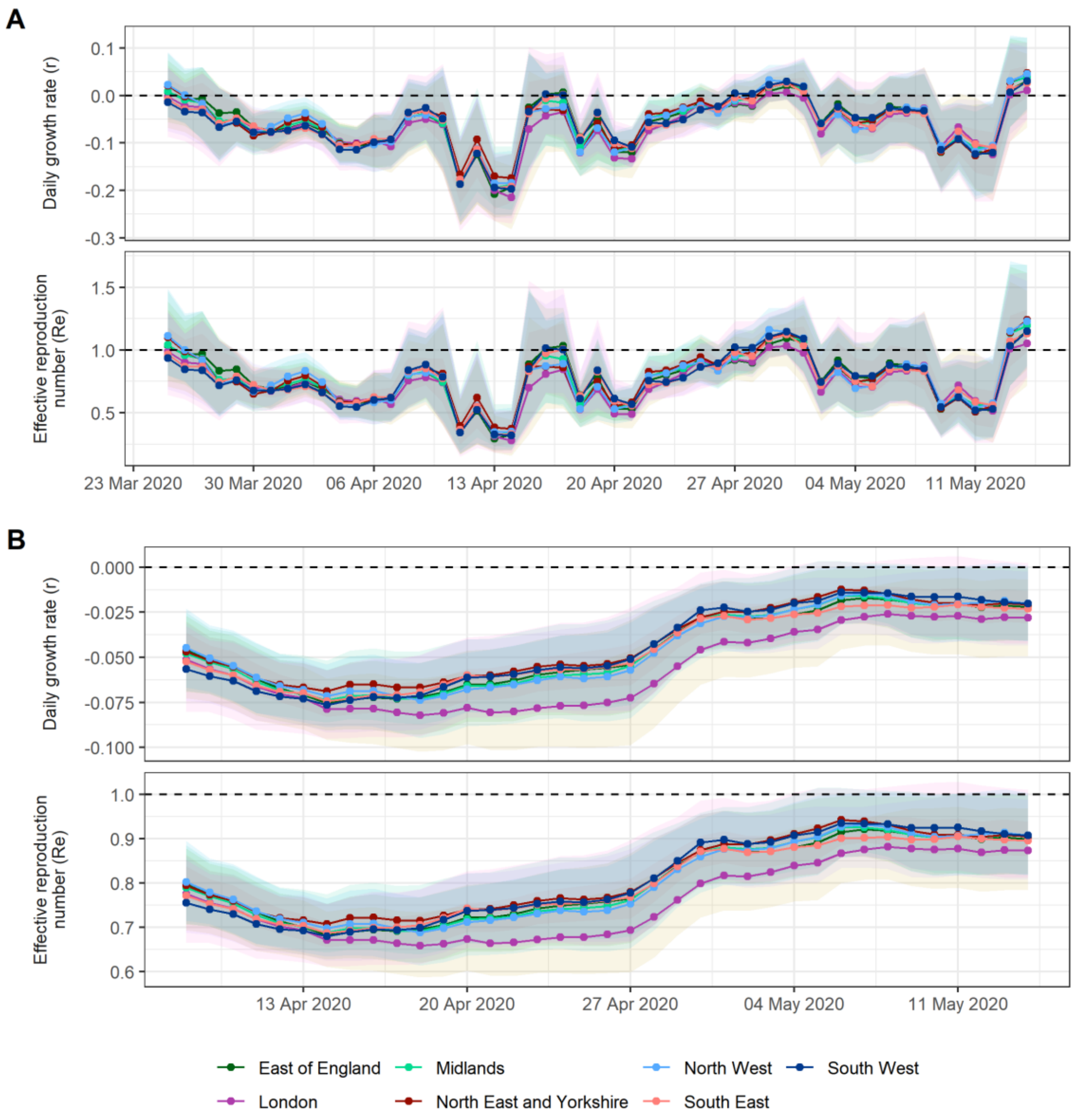

Figure S1. Estimates of daily growth rates (r) and effective reproduction numbers (R_e) for potential COVID-19 cases (all ages) reported through NHS Pathways. *Dotted lines indicate the central estimate, and ribbons their 95% confidence intervals. Estimates are indicated at the end of the time window used for estimation, so that values of r and R_e provided on a given day correspond to the chosen number of weeks leading up to that day. The size of the rolling window is A) 1 week; and B) 3 weeks.*

Serial interval distribution

We parameterised our serial interval distribution with a mean of 4.7 days and a standard deviation of 2.9 days. These parameters were obtained from a previous study by Nishiura et al [11], where the authors concluded that a lognormal distribution provided the best fit to their data. However, in our analysis the non-truncated lognormal distribution led to high serial interval values, with a maximum value greater than 200 days (Figure S2B), which heavily influenced our R_e estimates. We therefore chose to use a gamma distribution instead, which generated a more appropriate range of serial interval values for our analysis (Figure S2A).

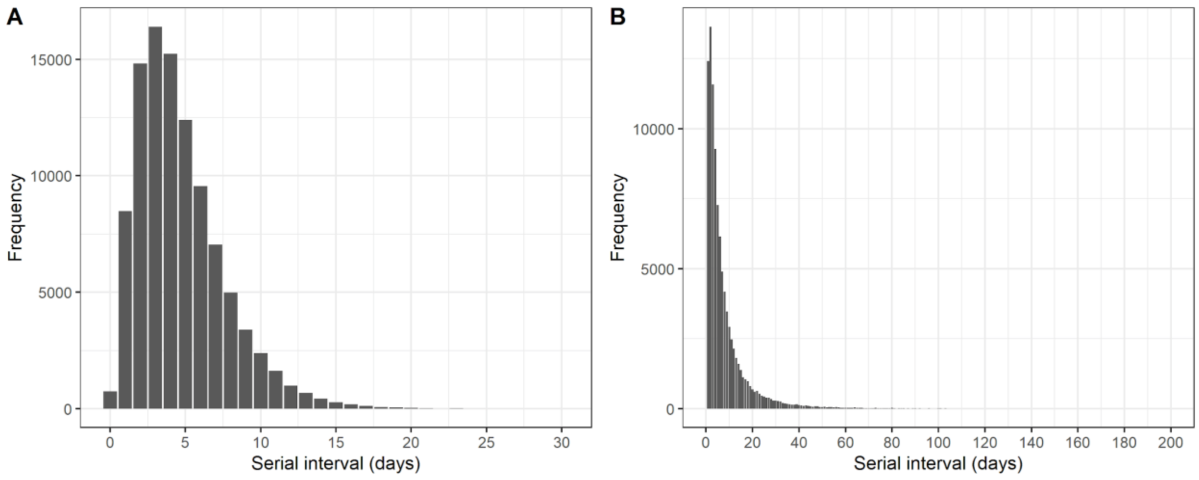

Figure S2. Serial interval distributions considered for our analysis. A) Gamma distribution; B) Lognormal distribution. *Both distributions are parameterised with a mean of 4.7 days and a standard deviation of 2.9 days. Serial interval values above 200 are not plotted.*

Age patterns

Reports are classified into 3 age groups in the NHS Pathways dataset: 0-18, 19-69 and 70-120 years old. A majority of all reports (86%) came from individuals between 19 and 69 years old (Figures S3 and S4). Despite some temporal variation, the proportion of cases from this age group exceeded proportions in the general population in all NHS regions (Figure S4). The proportion of reports of potential cases aged 0-18 years old decreased rapidly in all regions from mid-March (about 20%) to early April (about 10%). We note that the proportion of reports for 0-18 years old was lowest between the 9th and 23rd April, which corresponds to the period where reports from this age group were not included in the 111-online subset of the data. However, since this age group represents a minority of total reports, and reports for other age categories were also decreasing at that time (Figure S5), this lack of reporting is likely not responsible for the overall negative growth rates we presented in our results.

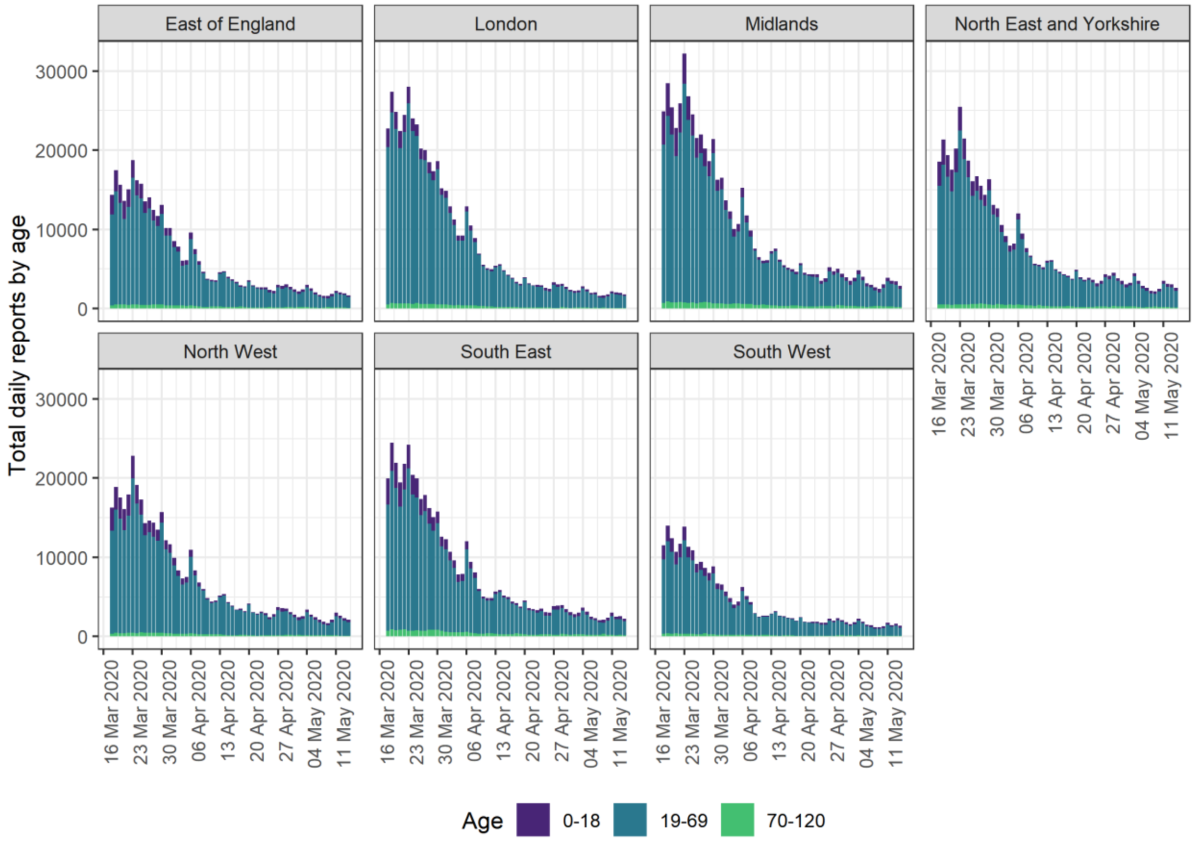

Figure S3. Daily potential COVID-19 cases reported through NHS Pathways, by NHS region and age group. *Data include calls to 111 and 999, as well as 111-online reports. Dates correspond to the date of case report, with x-axis labels corresponding to Mondays.*

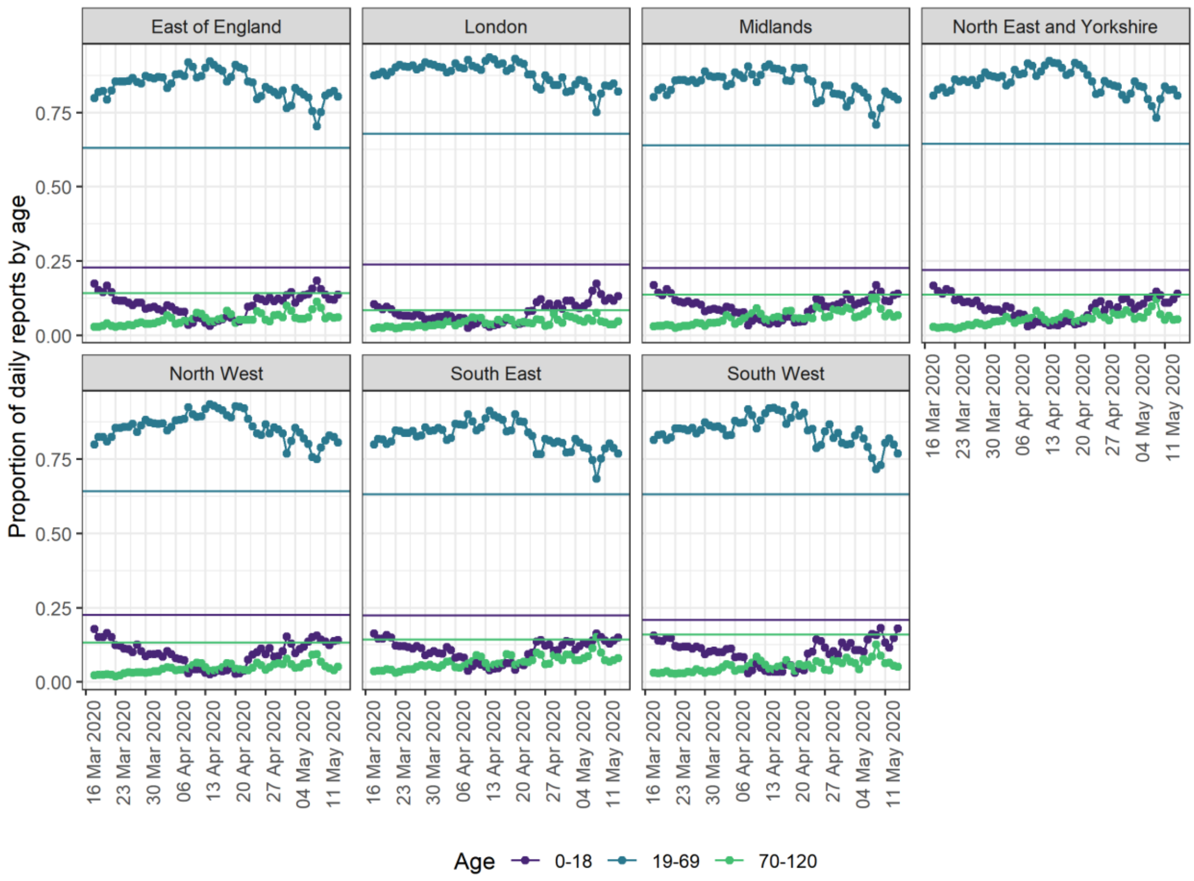

Figure S4. Proportion of potential COVID-19 cases reported through NHS Pathways by age group. *Dates correspond to dates of report. Proportions are derived from all potential COVID-19* reports *including 111 and 999 calls, and 111-online. Horizontal, solid lines represent the age distribution for the total population of the corresponding NHS region.*

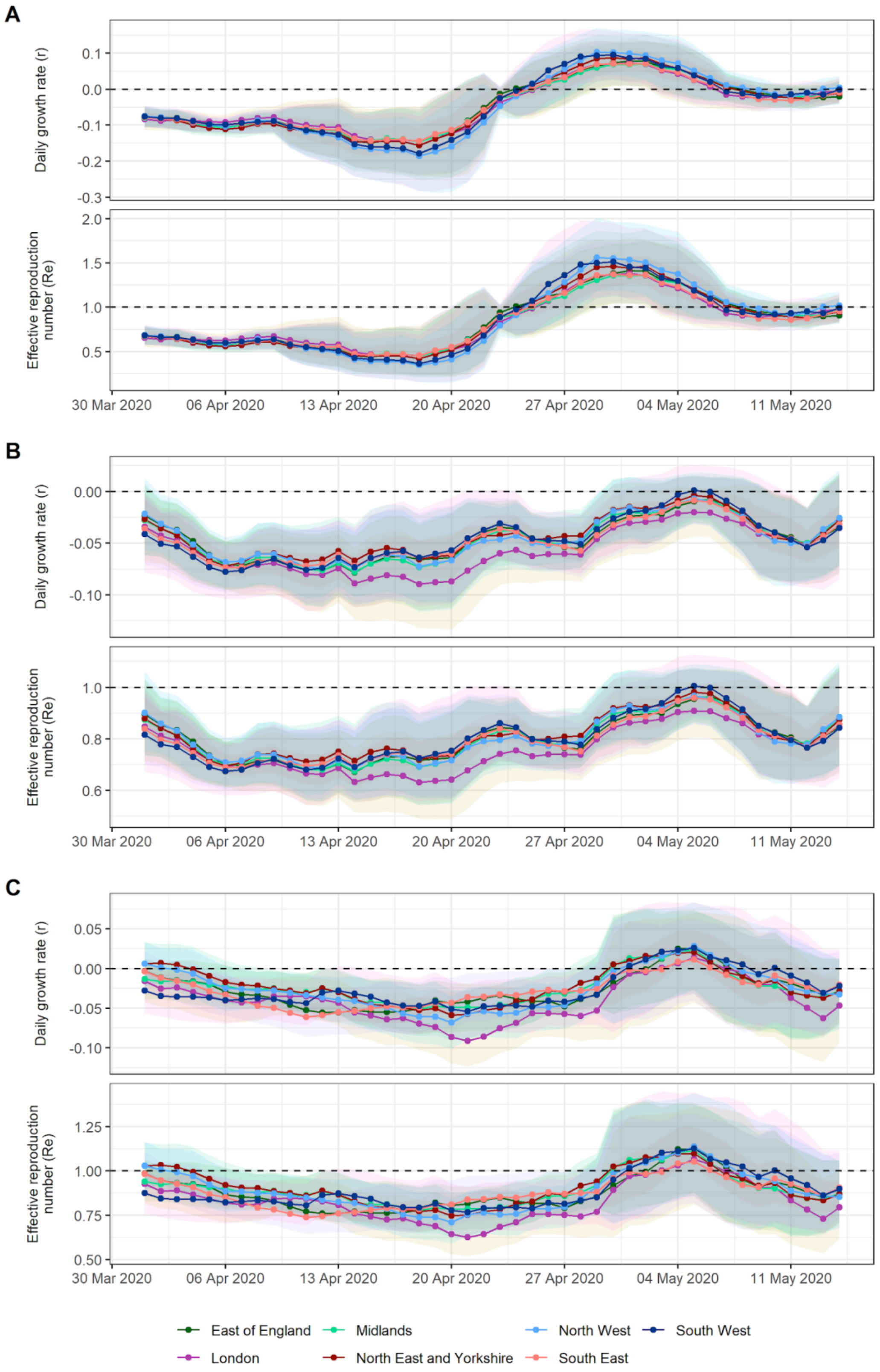

***Figure S5. Estimates of daily growth rates (r) and effective reproduction number (R_e) for potential COVID-19 cases reported through NHS Pathways, stratified by age**. A) Cases aged 0 to 18 years old; B) Cases aged 19 to 69 years old; C) Cases aged 70 to 120 years old. Lines and points indicate the central estimate, and ribbons their 95% confidence intervals. Estimates are indicated at the end of*

the time window used for estimation, so that values of r and R_e provided on a given day correspond to the 2 weeks leading up to that day.

Correlation between NHS Pathways reports and deaths by region

We repeated the correlation analysis between NHS Pathways reports and deaths for each region separately. The values suggest that the correlation reported in the main text for England overall could also potentially hold true for trends seen at the regional level, but the corresponding lag time would vary by a few days between regions (Figure S6). At this stage however, we cannot conclude this with certainty, as the correlation values are further from 1 than the one we report in the main text for England overall.

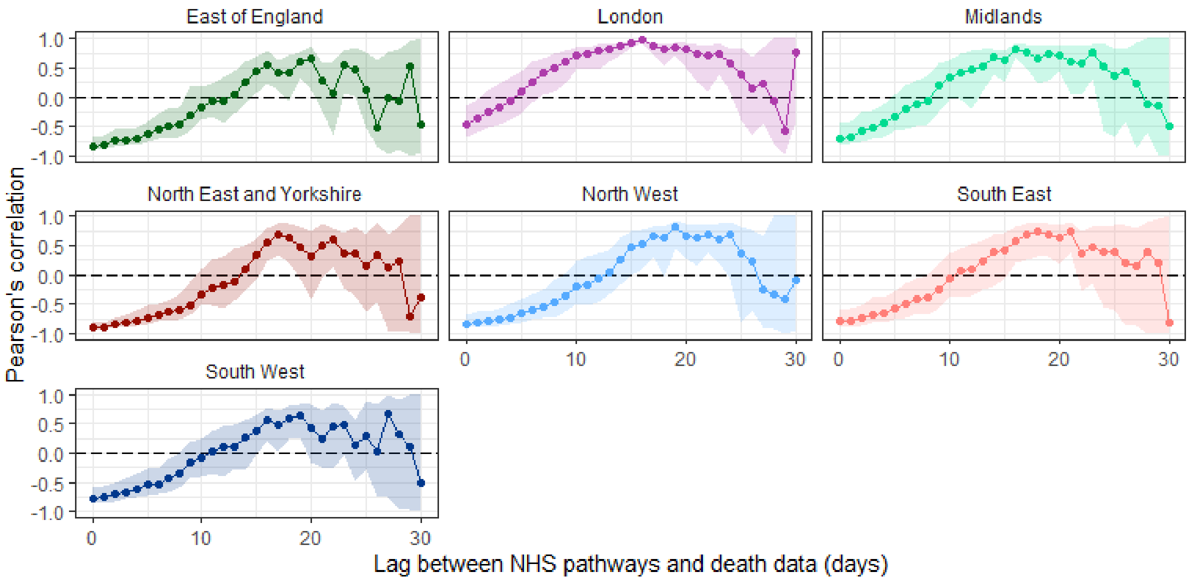

Figure S6. ***Pearson's correlation between deaths and potential COVID-19 cases reported through NHS Pathways, lagged between 0 and 30 days and separated by NHS regions.*** *95% confidence intervals are calculated by bootstrapping with 1,000 replicates.*
